# Age and sex affect the association of systolic blood pressure with clinical outcomes in thrombolysed stroke patient: a secondary analysis of the INTRECIS study

**DOI:** 10.3389/fneur.2023.1273131

**Published:** 2023-10-18

**Authors:** Bai-Jun Liu, Jing Li, Hui-Sheng Chen

**Affiliations:** Department of Neurology, General Hospital of Northern Theater Command, Shenyang, Liaoning, China

**Keywords:** age, sex, systolic blood pressure, ischemic stroke, thrombolysis, outcome

## Abstract

**Background and purpose:**

Blood pressure is associated with outcomes in acute ischemic stroke (AIS) patients receiving intravenous alteplase. The study aimed to explore the effect of sex and age on their association.

**Methods:**

Based on a prospective cohort, we retrospectively enrolled consecutive AIS patients who received intravenous alteplase and had complete blood pressure data, including baseline systolic blood pressure (SBP 01), SBP at 1 h (SBP 02), and SBP at 24 h (SBP 03) after alteplase. Maximum SBP (SBP max), minimum SBP (SBP min), and mean SBP (SBP mean) were calculated. Poor outcome was defined as having a modified Rankin Scale (mRS) score of 2–6 at 90 days. We explored the effect of age and sex on the association of different SBP indicators with the 3-month outcomes.

**Results:**

A total of 1,593 eligible patients were included in the present study. All SBP indicators were found to be higher in patients with poor vs. good outcomes. Multivariate logistic regression analysis showed that all SBP indicators except baseline SBP were associated with poor outcomes with good prediction powers (AUC, 0.762–0.766). More SBP indicators (SBP 02, SBP 03, SBP min, and SBP mean) were associated with poor outcomes in women vs. men, while all SBP indicators after alteplase were associated with poor outcomes in patients aged ≥ 60 years, but none was seen in patients aged < 60 years. Furthermore, all SBP indicators after alteplase were associated with poor outcomes in women aged ≥ 60 years, while only SBP 03 in men aged < 60 years.

**Conclusion:**

Among Chinese stroke patients treated with intravenous alteplase, SBP after alteplase was associated with clinical outcomes, which were affected by age and sex.

## Introduction

Intravenous thrombolysis (IVT) is an approved effective treatment for acute ischemic stroke (AIS), but many patients still achieved poor outcomes (1). Many factors were associated with poor outcomes, in which blood pressure was closely associated with poor outcomes (2). Three quarters of AIS patients had elevated blood pressure at presentation with about half of them with a history of hypertension (3). The elevated blood pressure in AIS patients was considered a compensatory response that would increase the perfusion of the ischemic cerebral tissue to save penumbra, whereas excessively elevated blood pressure would worsen cerebral edema and result in hemorrhagic transformation, especially for patients with IVT (4). Some studies suggested that higher mean systolic blood pressure (SBP) (5, 6), greater SBP variability (7–9), and smaller reductions in SBP (8, 9), after IVT were associated with poor outcomes. In AIS patients, a decrease in SBP may reduce the risk of hemorrhagic transformation but decrease perfusion in the penumbra, leading to a poor outcome (10). Current clinical guidelines consistently recommend that SBP should be controlled below 185 mmHg in thrombolysed AIS patients (11).

The age and sex differences in stroke have been widely investigated. Women differed from men in the distribution of risk factors, stroke severity, and outcome, even in lacunar infarcts (12–15). Recent studies found significant sex differences in the clinical outcomes of stroke patients, such as endovascular treatment (16, 17) and intravenous thrombolysis (18, 19). In addition to sex, age was closely related to stroke outcomes, and increased age is often closely linked with poor function recovery (20–22). Collectively, these studies suggest the important effect of sex and age on stroke prevention, prognosis prediction, and treatment strategy. However, the effect of age and sex on the association of SBP with outcomes in IVT-treated patients is not well established.

In this context, we hypothesize that age and sex may influence the association of SBP with clinical outcomes in AIS patients after IVT, which was investigated by a *post hoc* analysis of the Intravenous Thrombolysis Registry for Chinese Ischemic Stroke within 4.5 h onset (INTRECIS) dataset.

## Methods

### Study design/patient population

The INTRECIS is a nationwide, multi-center, prospective, and registry study of consecutive adult AIS patients who received intravenous thrombolysis within 4.5 h of the onset of symptoms. The details of the study design have been reported recently (23). From the INTRECIS cohort, patients were included in the current study with the following criteria: (1) consecutive adult AIS patients who received 0.9 mg/kg intravenous alteplase within 4.5 h of a definite time of onset of symptoms; (2) the complete clinical data including SBP at baseline, 1, and 24 h after alteplase. Patients were excluded if they met the following criteria: (1) patients who received urokinase; (2) patients who received a non-standard dose of alteplase; (3) patients who lacked complete clinical data; (4) age > 80 years; (5) patients treated with mechanical thrombectomy. All patients and/or their legally authorized surrogates gave written informed consent.

We collected baseline characteristics of patients including age, sex, current smoker, current drinker, hypertension, diabetes mellitus, history of stroke, coronary heart disease, atrial fibrillation, body mass index, baseline heart rate, symptom onset-to-thrombolysis time (OTT), door-to-needle time (DNT), National Institute of Health Stroke Scale (NIHSS) score, blood pressure data including diastolic blood pressure, baseline SBP (SBP01), immediate SBP after the end of alteplase (SBP02), SBP 24 h after alteplase (SBP03), maximum SBP among three timepoints (SBP max), minimum SBP among three timepoints (SBP min), and average SBP among three timepoints (SBP mean), and mRS at 90 days. Blood pressure was measured using a validated electronic sphygmomanometer while the patient was supine.

### Clinical outcomes

In parallel with the INTRECIS study (23), the poor outcome was defined as a mRS score of 2–6 points at 90 days, whereas the good outcome was defined as a mRS score of 0–1 points at 90 days.

### Statistical analysis

We performed descriptive statistics for baseline characteristics. Baseline information was compared between favorable and poor outcome groups using the *t*-test or *U*-test for continuous variables and the chi-square test or Fisher's exact test for categorical variables. First, we performed the univariate logistic regression analysis to identify the associated variables with poor outcomes. In the multivariate logistic regression analysis, BMI, baseline NIHSS score, baseline heart rate, current drinker, previous stroke, and atrial fibrillation were further adjusted to determine whether different SBP metrics were associated with patient outcomes after thrombolysis. Results are reported as odds ratios (OR) and 95% confidence intervals (CI). Differences with *P*-values < 0.05 were considered statistically significant in the relevant analytical tests. Second, we used a receiver operating characteristic (ROC) curve to explore the predictive value of SBP *via* areas under the curve (AUCs). The statistical software SPSS version 26.0 was used for analysis.

## Results

### Baseline data

**Figure 1:**
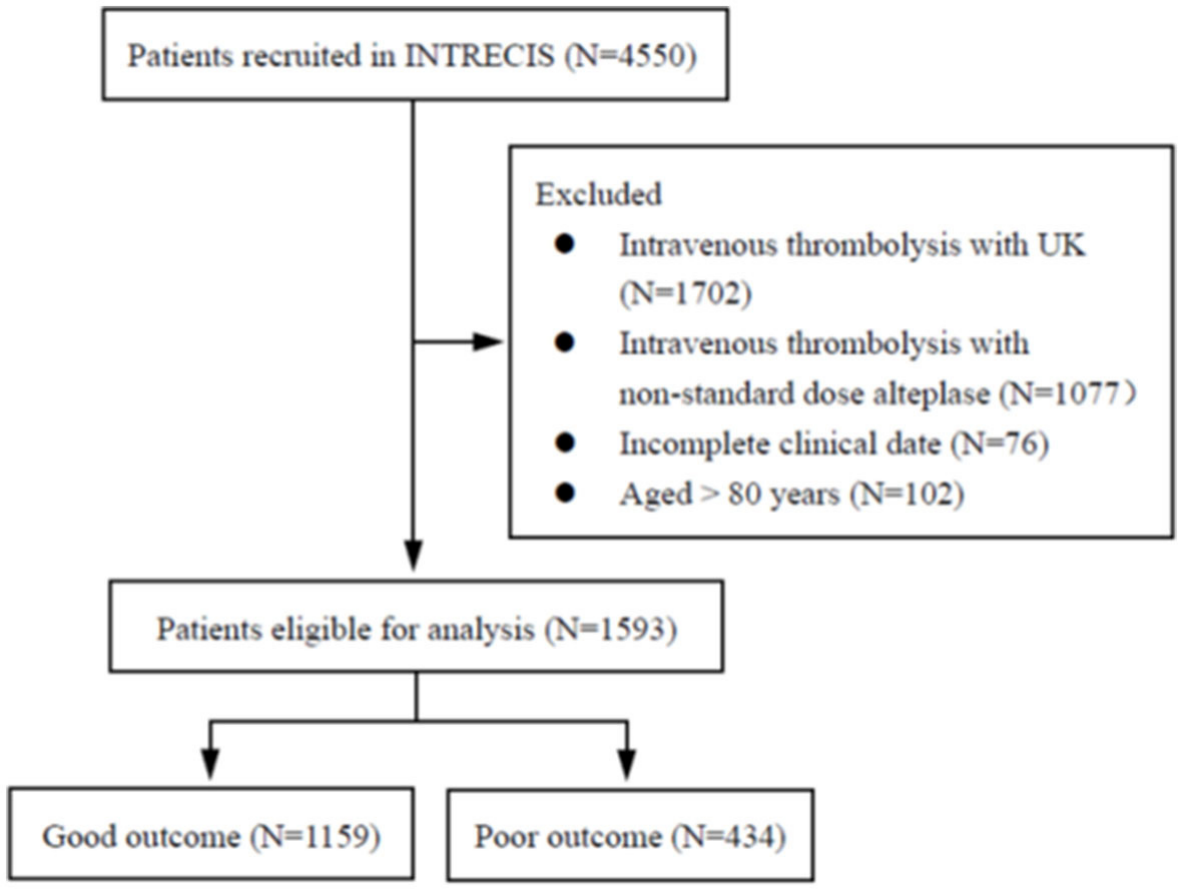
Flow chart of eligible patients.

From 4,550 patients enrolled in the INTRECIS cohort between October 2016 and September 2019, 1,593 eligible patients were included in this study: 1,159 (72.8%) in the good outcome group and 434 (27.2%) in the poor outcome group (Figure 1). Table 1 presents baseline characteristics in two groups. Patients with poor outcomes were older (65 years vs. 62 years, *P* < 0.000), had higher NIHSS score at admission (11 vs. 5, *P* < 0.000), and higher SBP indicators such as median SBP02 (145 mmHg vs. 141 mmHg, *P* = 0.010), median SBP03 (142 mmHg vs. 140 mmHg, *P* < 0.000), median SBP max (156 mmHg vs. 154 mmHg, *P* = 0.012), and median SBP min (137.5 mmHg vs. 135 mmHg, *P* = 0.008), and median SBP mean (146.7 mmHg vs. 143.7 mmHg, *P* = 0.004) (Table 1).

**Table 1:** Baseline characteristics in good outcome and poor outcome groups.

**Variable**	**Total (*n =* 1,593)**	**Good outcome (*n =* 1,159)**	**Poor outcome (*n =* 434)**	***P*–value**
Age, y	62 (54–69)	62 (54–68)	65 (56.8–72)	0.000
Men	1,113 (69.9%)	823 (71.0%)	290 (66.8%)	0.105
Hypertension	890 (55.9%)	635 (54.8%)	255 (58.8%)	0.156
Diabetes mellitus	303 (19.0%)	220 (19.0%)	83 (19.1%)	0.949
History of stroke	304 (19.1%)	201 (17.3%)	103 (23.7%)	0.004
Coronary heart disease	218 (13.7%)	149 (12.9%)	69 (15.9%)	0.116
Atrial fibrillation	138 (8.7%)	79 (6.8%)	59 (13.6%)	0.000
Current smoker	626 (39.3%)	463 (39.9%)	163 (37.6%)	0.384
Current drinker	384 (24.1%)	299 (25.8%)	85 (19.6%)	0.010
**TOAST classification**				0.262
LAA, *n* (%)	781 (49.0%)	525 (45.3%)	256 (59.0%)	
CE, *n* (%)	149 (9.4%)	92 (7.9%)	57 (13.1%)	
SAO, *n* (%)	516 (32.4%)	436 (37.6%)	80 (18.4%)	
ODC, *n* (%)	39 (2.4%)	27 (2.3%)	12 (2.8%)	
UND, *n* (%)	108 (6.8%)	79 (6.8%)	29 (6.7%)	
BMI (kg/m^2^)	23.9 (21.5–26.1)	24 (21.5–26.2)	23.4 (21.4–25.9)	0.031
Baseline heart rate	76 (69–85)	76 (69–84)	78 (69.8–86)	0.058
OTT (min)	170 (130–210)	171 (131–210)	170 (127.8–210)	0.366
DNT (min)	58 (38–85)	57 (37–85)	60 (39.8–90)	0.220
Baseline NIHSS	6 (3–10)	5 (3–8)	11 (6–15)	0.000
SBP01 (mmHg)	150 (136–165.5)	150 (135–165)	152 (138–167)	0.257
SBP02 (mmHg)	142 (131–154)	141 (130–152)	145 (134.8–156)	0.010
SBP03 (mmHg)	140 (130–150)	140 (129–148)	142 (130–153.3)	0.000
SBP max (mmHg)	155 (141–168)	154 (140–167)	156 (143–170.1)	0.012
SBP min (mmHg)	135 (124–145)	135 (124–144)	137.5 (124.8–147)	0.008
SBP mean (mmHg)	144.3 (134.3–155.3)	143.7 (134–154)	146.7 (135.7–158)	0.004
DBP (mmHg)	89 (80–98)	89 (80–98)	88 (80–99)	0.924
Any ICH within 24 h	15	10 (0.8)	5 (1.1)	0.242
sICH within 24 h	16	3 (0.2)	13 (2.7)	0.000

Values are n/N (%) or median (IQR) unless otherwise as indicated. IQR, interquartile range; NIHSS, National Institutes of Health Stroke Scale; SBP, systolic blood pressure; DBP, diastolic blood pressure; SBP01, baseline SBP; SBP02, SBP at 1 h; SBP03, SBP at 24 h; SBP max, maximum SBP among three timepoints; SBP min, minimum SBP among three timepoints; SBP mean, average of SBP among three timepoints; BMI, body mass index; OTT, onset-to-treatment time; DNT, door-to-needle time.

**Figure 2:**
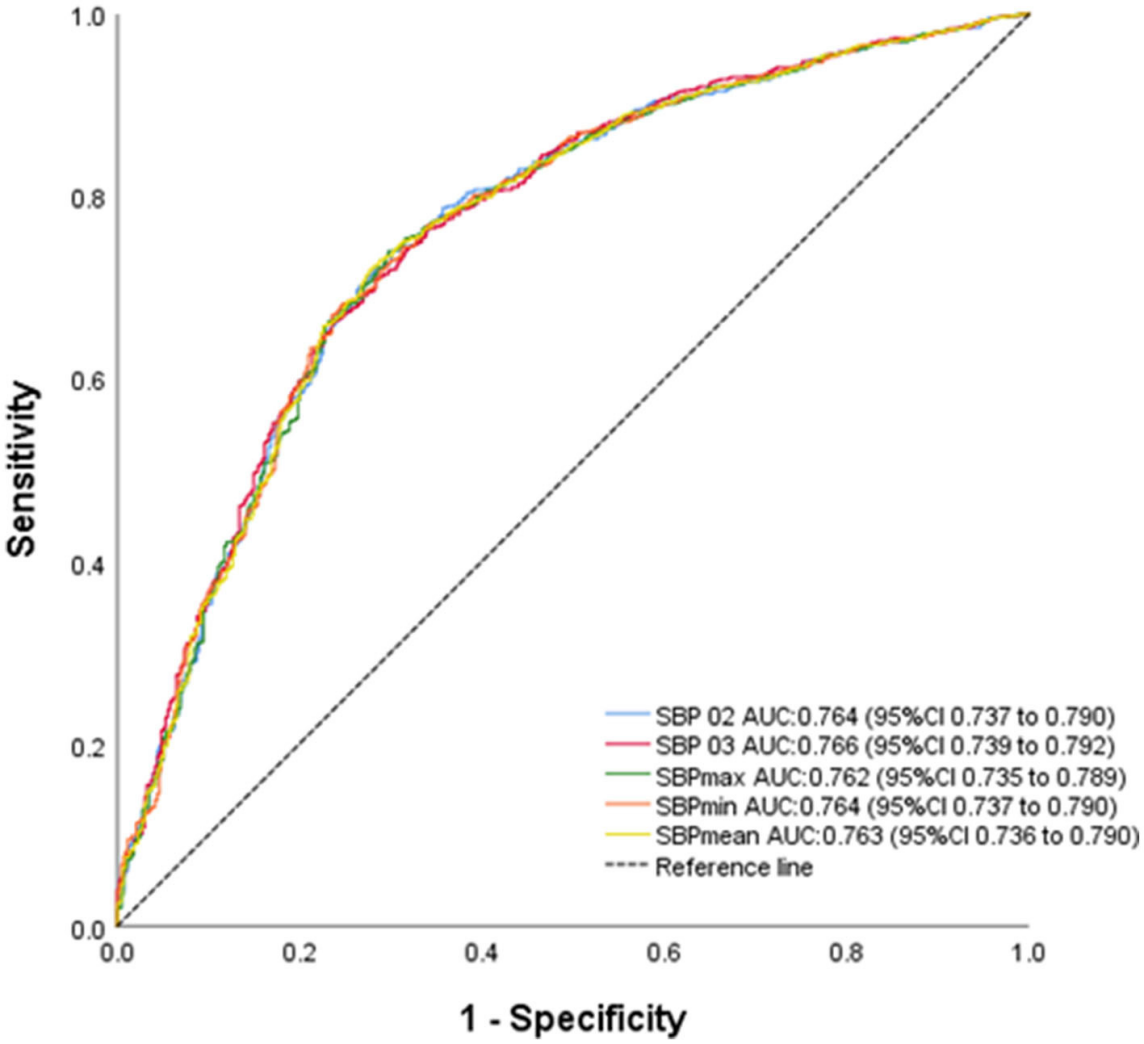
Receiver operating characteristic curve of prediction of functional outcomes.

In the univariate logistic regression analysis, age, body mass index, current drinker, history of stroke, atrial fibrillation, baseline heart rate, NIHSS score at baseline, SBP02, SBP03, SBP max, SBP min, and SBP mean were statistically significant (*P* < 0.05, Table 2). In multivariate logistic regression analysis (Table 2), SBP02, SBP03, SBP max, SBP min, and SBP mean after IVT were associated with a higher likelihood of good outcomes after adjusting for variables with *P* < 0.05 in univariate regression analysis (per 10 mm Hg higher SBP indicator, adjusted OR 0.87–0.94, all *P* < 0.05). Furthermore, ROC curve analysis showed these SBP indicators had good prediction power for functional outcomes: SBP02 with an AUC of 0.764 (95% CI = 0.737–0.790), SBP03 with an AUC of 0.766 (95% CI = 0.739–0.792), SBP max with an AUC of 0.762 (95% CI = 0.735–0.789), SBP min with an AUC of 0.764 (95% CI = 0.737–0.790), and SBP mean with an AUC of 0.763 (95% CI = 0.736–0.790) (Figure 2).

**Table 2:** Univariate and multivariate logistic regression analysis for good outcomes.

**Variable**	**Unadjusted OR (95% CI)**	***P*–value**	Adjusted^*^OR (95% CI) ^#^	***P*–value**	**PPV**	**NPV**	**Diagnostic accuracy**
Age	0.968 (0.957–0.979)	< 0.001					
BMI	1.034 (1.003–1.066)	0.031					
Door–to–needle time	0.999 (0.996–1.002)	0.413					
Onset–to–treatment time	1.001 (0.999–1.003)	0.271					
Men	0.822 (0.649–1.042)	0.105					
Current smoker	1.106 (0.881–1.388)	0.385					
Current drinker	1.428 (1.008–1.872)	0.010					
Hypertension	0.851 (0.680–1.064)	0.156					
Coronary heart disease	0.780 (0.573–1.063)	0.116					
Previous stroke	0.674 (0.516–0.882)	0.004					
Diabetes mellitus	0.991 (0.748–1.312)	0.949					
Atrial fibrillation	0.465 (0.325–0.664)	< 0.001					
Baseline NIHSS	0.858 (0.840–0.877)	< 0.001					
Baseline heart rate	0.989 (0.982–0.997)	0.006					
SBP 01	0.975 (0.929–1.024)	0.307					
SBP 02	0.918 (0.861–0.979)	0.009	0.910 (0.848–0.976)	0.008	59.9%	77.6%	75.3%
SBP 03	0.868 (0.813–0.928)	< 0.001	0.870 (0.810–0.935)	< 0.001	62.0%	77.9%	75.8%
SBP max	0.934 (0.885–0.985)	0.012	0.940 (0.887–0.997)	0.040	61.4%	77.9%	75.8%
SBP min	0.911 (0.850–0.977)	0.009	0.899 (0.833–0.971)	0.007	62.0%	75.5%	75.9%
SBP mean	0.904 (0.844–0.968)	0.004	0.902 (0.837–0.973)	0.007	60.3%	77.8%	75.5%

OR, odds ratio; CI, confidence interval; BMI, body mass index; SBP, systolic blood pressure; SBP01, baseline SBP; SBP02, SBP at 1 h; SBP03, SBP at 24 h; SBP max, maximum SBP among three timepoints; SBP min, minimum SBP among three timepoints; SBP mean, average of SBP among three timepoints; PPV, positive predictive value; NPV, negative predictive value.

* Adjusted for body mass index, baseline NIHSS score, baseline heart rate, current drinkers, previous stroke, and atrial fibrillation, SBP 02, SBP 03, SBP max, SBP min, and SBP mean.

# OR per 10 mm Hg increase in SBP indicator measure.

Based on sex, patients were divided into two groups: 1113 in the men group and 480 in the women group. After adjusting for BMI, baseline NIHSS score, baseline heart rate, current drinker, previous stroke, and atrial fibrillation, the multivariate logistic regression analysis showed that all SBP indicators after IVT except SBP max were associated with outcome in women, while only SBP03 in men (Table 3).

**Table 3:** Logistic regression analysis of SBP indicators on poor outcomes stratified by sex.

**Variable**	**Men**			**Women**		
	**Median, IQR**	**Adjusted** ^*^OR (95% CI) ^#^	***P*** **–value**	**Median, IQR**	**Adjusted** ^*^OR (95% CI) ^#^	***P**−**0*** **value**
SBP 02	140 (130–152)	1.085 (0.998–1.178)	0.055	145 (132–154)	1.147 (1.000–1.314)	0.049
SBP 03	139 (127–149)	1.138 (1.043–1.241)	0.004	140 (130–148)	1.190 (1.043–1.357)	0.010
SBP max	152 (140–166)	1.046 (0.974–1.123)	0.215	156 (143–168)	1.113 (1.000–1.239)	0.051
SBP min	134 (124–143)	1.095 (0.999–1.200)	0.052	136 (126–145)	1.165 (1.008–1.346)	0.039
SBP mean	143 (133–153)	1.084 (0.991–1.186)	0.079	146 (136–156)	1.185 (1.027–1.367)	0.020

OR, odds ratio; CI, confidence interval; SBP, systolic blood pressure; SBP02, SBP at 1 h; SBP03, SBP at 24 h; SBP max, maximum SBP among three timepoints; SBP min, minimum SBP among three timepoints; SBP mean, average of SBP among three timepoints.

* Adjusted for body mass index, baseline NIHSS score, baseline heart rate, current drinkers, previous stroke, and atrial fibrillation, SBP 02, SBP 03, SBP max, SBP min, and SBP mean.

# OR per 10 mm Hg increase in SBP indicator measure.

**Figure 3:**
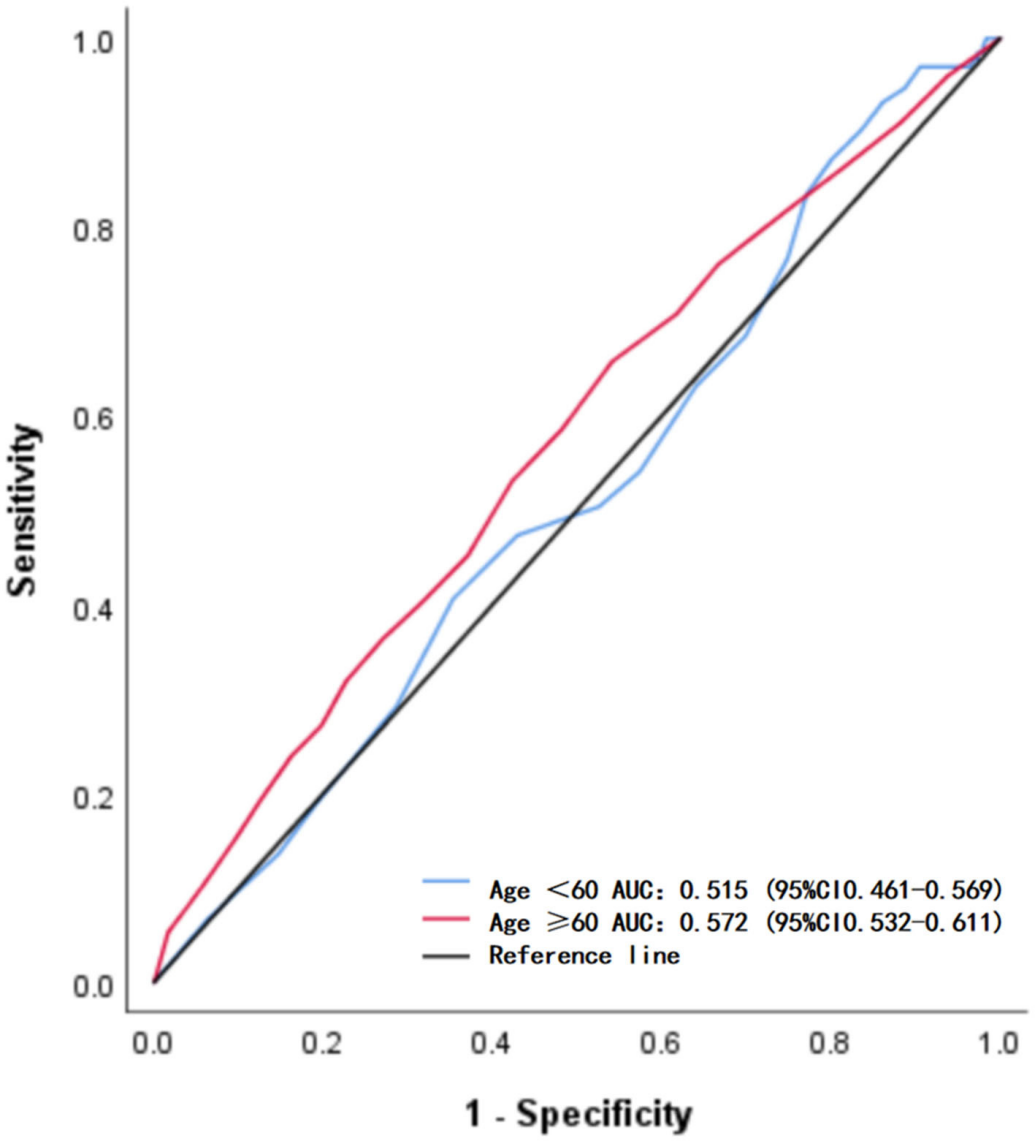
Receiver operating characteristic curves for predicting functional outcomes in two age subgroups.

According to the age, patients were classified into two groups: 962 in the ≥ 60-year-old group and 631 in the < 60-year-old group. After adjusting for BMI, baseline NIHSS score, baseline heart rate, current drinker, previous stroke, and atrial fibrillation, the multivariate logistic regression analysis showed that all SBP indicators after IVT were associated with outcomes in the ≥ 60 years group, but none were associated in the < 60 years group (Table 4). Furthermore, ROC curve analysis showed age had moderate prediction power for functional outcomes: ≥ 60 years with an AUC of 0.572 (95% CI = 0.532–0.611) and < 60 years with an AUC of 0.515 (95% CI = 0.461–0.569) (Figure 3).

**Table 4:** Logistic regression analysis of SBP indicators on poor outcomes stratified by age.

**Variable**	**Men**			**Women**		
	**Median, IQR**	**Adjusted** ^*^OR (95% CI) ^#^	***P*** **–value**	**Median, IQR**	**Adjusted** ^*^OR (95% CI) ^#^	***P*** **–value**
SBP 02	142 (132–154)	1.113 (1.019–1.217)	0.018	140 (130–152)	1.057 (0.941–1.188)	0.350
SBP 03	140 (130–148)	1.164 (1.063–1.275)	0.001	138 (126–149)	1.106 (0.981–1.247)	0.100
SBP max	155 (141–168)	1.081 (1.005–1.163)	0.037	152 (140–166)	1.013 (0.915–1.122)	0.800
SBP min	135 (126–144)	1.120 (1.015–1.236)	0.024	133 (121–143)	1.075 (0.948–1.218)	0.259
SBP mean	145 (136–154)	1.128 (1.025–1.241)	0.014	133 (121–143)	1.052 (0.928–1.193)	0.429

OR, odds ratio; CI, confidence interval; SBP, systolic blood pressure; SBP02, SBP at 1 h; SBP03, SBP at 24 h; SBP max, maximum SBP among three timepoints; SBP min, minimum SBP among three timepoints; SBP mean, average of SBP among three timepoints.

* Adjusted for body mass index, baseline NIHSS score, baseline heart rate, current drinkers, previous stroke, and atrial fibrillation, SBP 02, SBP 03, SBP max, SBP min, and SBP mean.

# OR per 10 mm Hg increase in SBP indicator measure.

**Figure 4:**
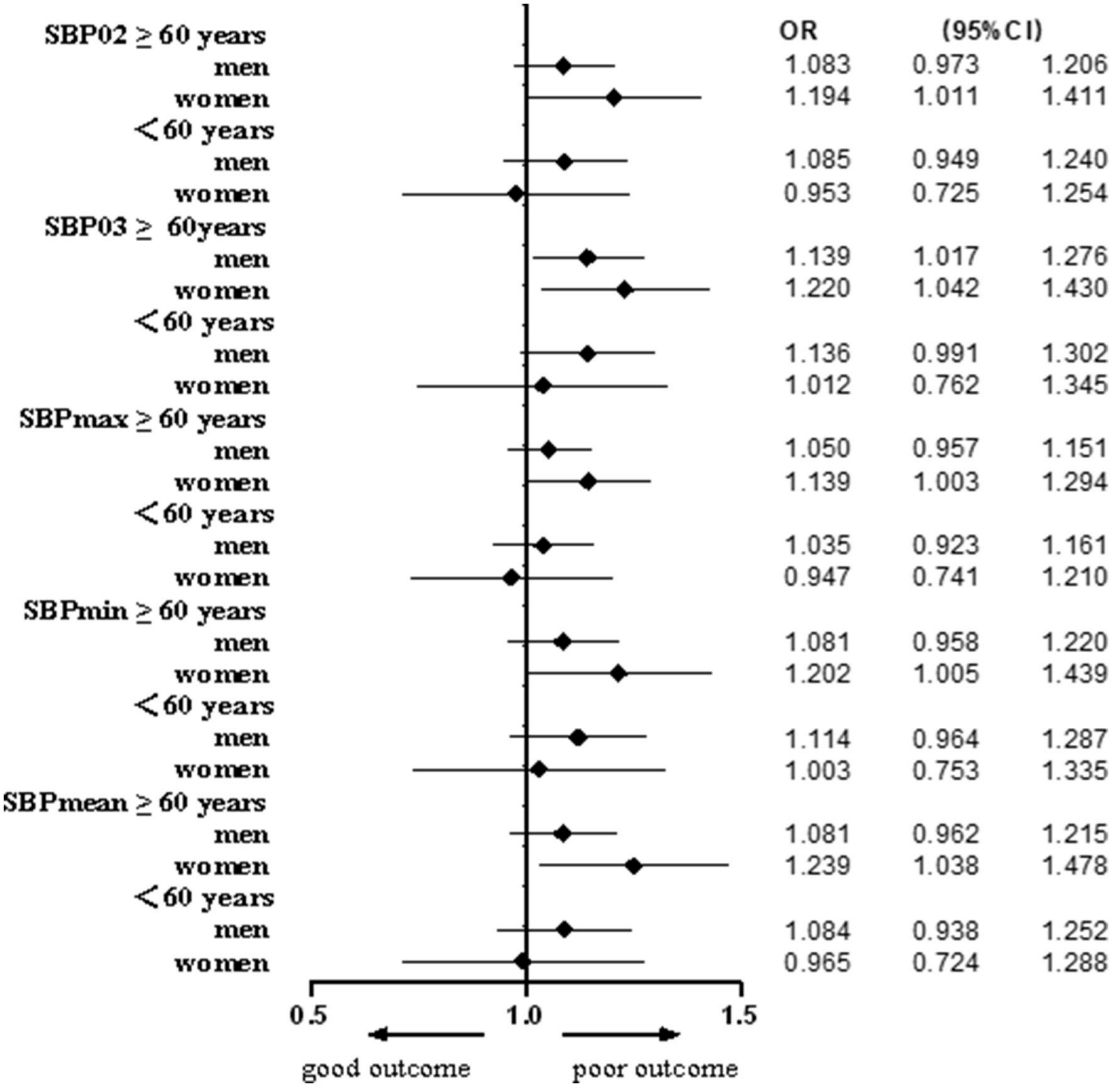
Forest plots of different SBP indicators predicting functional outcomes stratified by age and sex.

Based on age and sex, we further divided the patients into four subgroups: men ≥60 years, women ≥60 years, men <60 years, and women <60 years. After adjusting for BMI, baseline NIHSS score, baseline heart rate, current drinker, previous stroke, and atrial fibrillation, the multivariate logistic regression analysis showed that all SBP indicators after IVT were associated with outcomes in the ≥60 years women group, while only SBP 03 were associated in the ≥60 years men group (Table 5, Figure 4).

**Table 5:** Logistic regression analysis of SBP indicators on poor outcomes stratified by age and sex.

	**Age** ≥**60**						**Age < 60**					
	**Men (n** = **621)**			**Women (n** = **341)**			**Men (n** = **492)**			**Women (n** = **139)**		
**Variable**	**Median, IQR**	Adjusted^*^OR (95% CI) ^#^	***P*** **–value**	**Median, IQR**	Adjusted^*^OR (95% CI) ^#^	***P*** **–value**	**Median, IQR**	Adjusted^*^OR (95% CI) ^#^	***P–*** **value**	**Median, IQR**	Adjusted^*^OR (95% CI) ^#^	***P*** **–value**
SBP 02	141 (131–155)	1.083 (0.973–1.206)	0.144	145 (134–153)	1.194 (1.011–1.411)	0.036	140 (130–152)	1.085 (0.949–1.240)	0.232	142 (130–156)	0.953 (0.725–1.254)	0.731
SBP 03	140 (128–149)	1.139 (1.017–1.276)	0.025	140 (132–148)	1.220 (1.042–1.430)	0.014	138 (126–149)	1.136 (0.991–1.302)	0.068	139 (126–150)	1.012 (0.762–1.345)	0.932
SBP max	154 (140–167)	1.050 (0.957–1.151)	0.303	156 (145–169)	1.139 (1.003–1.294)	0.046	151 (140–166)	1.035 (0.923–1.161)	0.559	156 (140–168)	0.947 (0.741–1.210)	0.661
SBP min	135 (125–143)	1.081 (0.958–1.220)	0.205	136 (130–145)	1.202 (1.005–1.439)	0.044	132 (120–142)	1.114 (0.964–1.287)	0.143	134 (122–145)	1.003 (0.753–1.335)	0.986
SBP mean	144 (135–153)	1.081 (0.962–1.215)	0.190	146 (137–156)	1.239 (1.038–1.478)	0.017	142 (132–153)	1.084 (0.938–1.252)	0.276	145 (132–157)	0.965 (0.724–1.288)	0.811

OR, odds ratio; CI, confidence interval; SBP, systolic blood pressure; SBP02, SBP at 1 h; SBP03, SBP at 24 h; SBP max, maximum SBP among three timepoints; SBP min, minimum SBP among three timepoints; SBP mean, average of SBP among three timepoints.

* Adjusted for body mass index, baseline NIHSS score, baseline heart rate, current drinkers, previous stroke, and atrial fibrillation, SBP 02, SBP 03, SBP max, SBP min, and SBP mean.

# OR per 10 mm Hg increase in SBP indicator measure.

**Figure 5:**
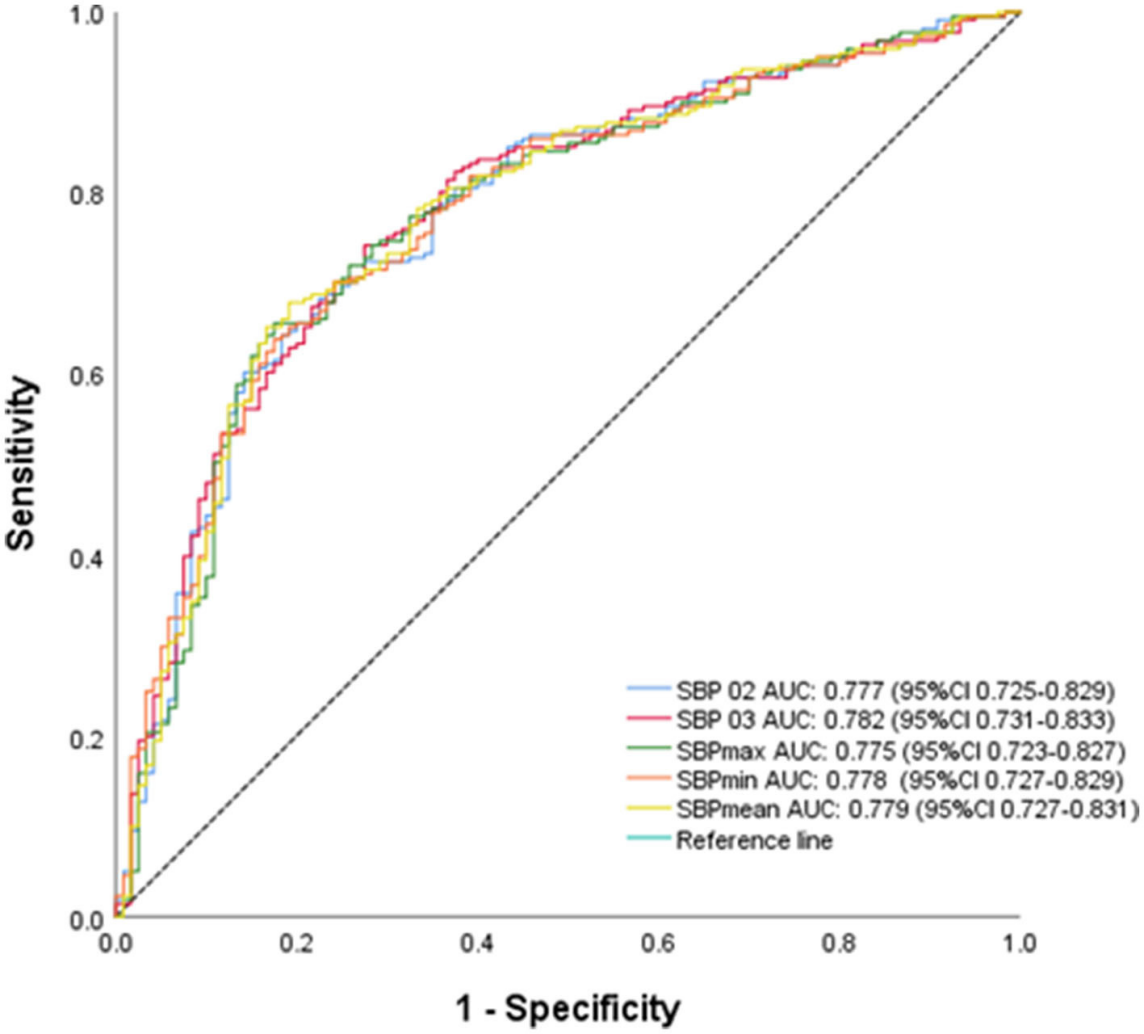
Receiver operating characteristic curve of prediction of functional outcomes in women patients ≥60 years.

Given the close association of SBP indicators with outcomes in the women subgroup with ≥ 60 years, ROC curve analysis was performed. The results showed SBP02 with an AUC of 0.777 (95% CI = 0.725–0.829), SBP03 with an AUC of 0.782 (95% CI = 0.731–0.883), SBP max with an AUC of 0.775 (95% CI = 0.723–0.827), SBP min with an AUC of 0.778 (95% CI = 0.727–0.829), and SBP mean with an AUC of 0.779 (95% CI = 0.727–0.839) (Figure 5).

### Discussion

In this *post hoc* analysis, we identified several findings: (1) Among AIS patients who received intravenous alteplase, SBP indicators after IVT, but not baseline SBP were associated with clinical outcomes; (2) more SBP indicators were associated with clinical outcomes in women but only SBP 03 in men; (3) the association of SBP indicators with clinical outcome was found in the ≥ 60 years group but not the < 60 years group; (4) in the ≥ 60 years group, more SBP indicators were associated with clinical outcomes in women but only SBP 03 in men. Collectively, these findings suggest that age and sex should influence the relationship between SBP and outcomes in thrombolysed patients, and the closer association of SBP with outcomes may be in the subgroup of women aged ≥ 60 years.

Prior studies have investigated the relationship between SBP levels and functional outcomes in thrombolysed AIS patients (24), but the results were inconsistent. In our study, there was no difference in SBP at baseline between good and poor outcome groups, but SBP at 1 h and 24 h after IVT was found to be significantly associated with clinical outcomes. The finding was in agreement with a previous study (7), but not with other studies reporting the close association of SBP at the baseline and up to 24 h after thrombolysis with poor prognosis (6, 8, 25). This conflicting reason was not clear. Given the potential effect of stress, bladder pressure, or other transient stimuli on SBP at the stroke onset, the association of baseline SBP with clinical outcomes should be complex. In summary, the current results suggested that increasing SBP after thrombolysis may reduce the likelihood of 3-month good functional outcomes.

The pathophysiological mechanism underlying their associations may be attributed to impaired cerebral autoregulation (CA) function following stroke. Under normal conditions, CA can maintain relatively constant cerebral blood flow (CBF) when arterial blood pressure or cerebral perfusion pressure fluctuates (26, 27). However, CA may be impaired or even vanish after ischemic stroke (28), which may partially explain the association of increased SBP with poor outcomes because impaired CA leads to ischemia-reperfusion injury or reduced cerebral perfusion (29, 30).

In the present study, we found the significant effect of age and sex on the relationship with SBP and functional outcomes in patients with AIS after IVT, and more closer association was identified in old women patients, which was never reported previously. These findings should be plausible. Compared to pre-menopausal women and age-matched men, post-menopausal women were found to have reduced vasomotor reserve, poorer cerebrovascular reactivity, and reduced cerebral blood flow autoregulation (31). Mechanisms underlying these changes may be related to estrogen. For example, estrogen can bind to estrogen receptors on the endothelium of cerebral arteries, which would cause vasodilatation in response to the deficit in cerebral perfusion (32). However, the protective effect of estrogen is lost in post-menopausal women, which makes them more susceptible to changes in blood pressure. As a non-modifiable risk factor for cerebrovascular diseases (33), aging would impair cerebral autoregulation (34). Collectively, these results suggest that aging and estrogen decrease in a synergistic manner cause the impairment of cerebral autoregulation, resulting in poor outcomes in this population.

The main strength of this study was the first attempt to determine the effect of sex and age on the association of SBP indicators with outcomes in patients with AIS after IVT. The results suggested that higher SBP indicators after IVT were associated with poor 3-month functional outcomes, especially in older women patients. However, we recognize several limitations. The main limitation was the retrospective analysis nature, which was subject to selection bias and unexpected confounding factors. Second, the current study was only performed in Chinese ischemic stroke patients, and patients over 80 years old were excluded due to too small sample size, which should limit the generalizability of the results. Third, the detailed data on antihypertensive drugs were not available, which may make it impossible to investigate their effects on the current findings. Fourth, given the sex difference in the distribution of risk factors, stroke severity, functional outcomes (12), the difference in the pathophysiology, prognosis, and clinical features of lacunar vs. non-lacunar stroke (35), and the relationship between SBP, sex, age, and outcomes warrants investigation in patients with lacunar vs. non-lacunar ischemic stroke. Fifth, patients with mild-to-moderate neurologic deficit (median NIHSS score of 6) were enrolled in this study, which limited the generalization in moderate and severe stroke. Sixth, the small absolute difference in SBP indicators in different groups would affect the practical implementation of this finding. Finally, these findings need to be explained with caution due to the exploratory nature of this secondary analysis.

In summary, among Chinese acute ischemic stroke patients, SBP indicators after intravenous thrombolysis were independently associated with outcomes at 3 months, which may be affected by age and sex. The relationship will be worth exploring in different stroke subtypes.

## Data availability statement

The raw data supporting the conclusions of this article will be made available by the authors, without undue reservation.

## Ethics statement

The studies involving humans were approved by the Institutional Review Board of General Hospital of Northern Theater Command (IRB: k2016–11). The studies were conducted in accordance with the local legislation and institutional requirements. The participants provided their written informed consent to participate in this study.

## Author contributions

B-JL: Data curation, Formal analysis, Writing—original draft. JL: Formal analysis, Writing—original draft. H-SC: Conceptualization, Funding acquisition, Project administration, Supervision, Writing—review and editing.
